# Recent Developments in HIV Antivirals: The Prospect of Prophylactic Drugs to Change the Pandemic

**DOI:** 10.1111/1751-7915.70317

**Published:** 2026-02-15

**Authors:** Harald Brüssow

**Affiliations:** ^1^ Laboratory of Gene Technology, Department of Biosystems KU Leuven Leuven Belgium

## Abstract

The human immunodeficiency‐1 virus was identified in 1983 as the etiological agent of AIDS. Four years later, the first HIV‐1 antiviral drug was approved: the nucleoside analog zidovudine inhibiting the viral reverse transcriptase (RT). Subsequently, non‐nucleoside inhibitors of RT, viral protease, viral integrase, and viral entry inhibitors were approved as antiviral drugs. Combining these drugs into a highly active antiretroviral therapy (HAART) decreased the viral load in chronically infected patients and suppressed AIDS defining symptoms. HAART became a medical success story, but the chronic HIV infection cannot be cured by antiviral drugs. Therefore, substantial efforts were undertaken to use antiviral drugs prophylactically to prevent HIV infections in high‐risk groups. PreExposure Prophylaxis (PrEP) with oral combined antiretroviral therapy (cART) showed some success in preventing infections in infants born to HIV‐infected mothers, decreased the rate of HIV infection in men having sex with men, in women having sex with men, in couples discordant for HIV status and in intravenous drug users. However, the daily burden of pill swallowing compromised seriously the adherence of people to antiviral drugs. The development of injectable long‐acting antiviral drugs based on the integrase inhibitor cabotegravir, which needs an injection every 2 months, or the viral capsid inhibitor lenacapavir, injected every half year, showed impressive results in prevention trials with men and women at high risk of infection. The present article describes aspects of HIV antiviral drug development, the outcome of pertinent clinical trials, and discusses economic and political hurdles for injected long‐acting antivirals to become a gamechanger for the HIV pandemic.

## Graphical abstract

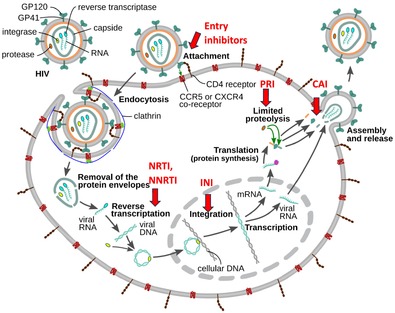

Scientific, medical and lay media have expressed substantial concern about the ongoing antibiotic resistance crisis. For several reasons, the problem with antiviral drugs is even more urgent. First, due to the high mutation rate in RNA viruses, resistant viruses even emerge in individual patients as seen in human immunodeficiency virus 1 (HIV‐1) infections treated with a single antiviral agent. Second, major viral pandemics such as AIDS and SARS‐CoV‐2 were caused by viruses that were only discovered recently: HIV‐1 in 1983 and SARS‐CoV‐2 in 2019. Third, only a few antiviral drugs were so far approved. Between 1959 and 2019, only 88 antiviral drugs were licensed including 43 against HIV‐1, 18 against hepatitis C virus (HCV), 11 against herpes viruses, 9 against influenza virus, and 8 against hepatitis B virus (Flint et al. 2020). Fourth, a further limitation to the development of antivirals is investment costs which can be as high as one billion US$. It therefore needs sufficiently large patient populations to make antiviral drug development commercially viable. This explains the focus on antivirals for chronic viral infections. Worldwide, the World Health Organization (WHO) estimated 37.7 million HIV‐infected and 170 million HCV‐infected people. Fifth, RNA viruses have small genomes providing only a few targets for antivirals. Sixth, targeting cellular functions that assist in viral propagation, as done by 13 approved antivirals, raises the problem of toxic side reactions and explains the high attrition rate in pharmaceutical development of antivirals (Flint et al. 2020). Finally, the accelerating rate of emerging viral epidemics over the last decades also calls for antivirals active against several classes of viruses, hopefully including the still unknown viral agent of a future pandemic (Lu et al. 2021) which is a tall challenge.

The present article will focus on HIV‐1 antivirals. They are a medical success story which transformed a formerly lethal viral disease into a chronic infection that can be managed by highly active antiretroviral therapy (HAART), which is a combined antiviral therapy (cART) with two or more antivirals to reduce the likelihood of viral resistance development under therapy. After shortly presenting the different anti‐HIV‐1 drug classes, the prophylactic use of antivirals to prevent HIV infection will be explored and the possibility will be discussed whether they could curtail the spread of the AIDS pandemic or even end it before developing an efficient vaccine.

## 1 HIV‐1 Antiviral Drug Classes Used in Antiviral Therapy

**FIGURE 1:**
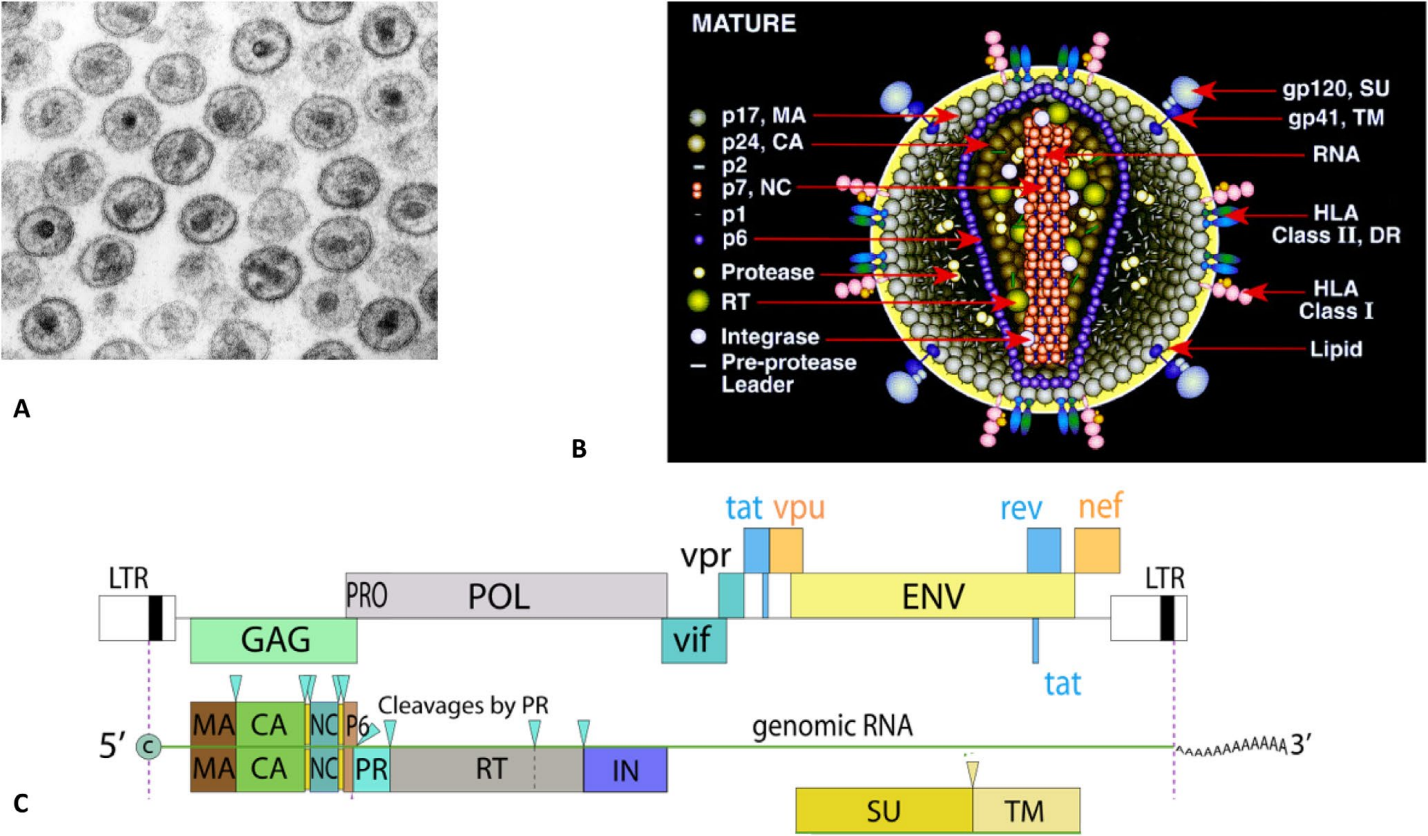
(A) Transmission electron micrograph of the 120 nm HIV‐1 particles. Cone‐shaped cores are sectioned in various orientations. Viral genomic RNA present in two copies is located in the electron‐dense wide end of the core. (B) Diagram of the mature HIV‐1 with location of its molecular components. Not all components are virus‐encoded. (C) Genetic organization of the 9.7 kb long HIV‐1 RNA genome. The three primary translation products from the *gag*, *pol* and *env* genes are synthesised as polyprotein precursors that are subsequently processed by proteases into the mature particle‐associated proteins. The 55‐kDa GAG (group‐specific antigen) precursor protein is cleaved into the matrix (MA), capsid (CA) nucleocapsid (NC) and p6 protein. Autocatalysis of the 160 kDa GAG‐POL polyprotein gives rise to the protease (PR), the reverse transcriptase (RT) consisting of the heterodimer p66 and p51 proteins, and the integrase (IN) p32. The proteolytic cleavage sites are indicated with sharp triangles above the gene map. The glycosylated 160 kDa ENV (envelope) precursor protein is converted into the gp120 surface (SU) and gp41 transmembrane (TM) cleavage products. Vif, Vpr, Tat, Rev, Vpu and Nef are primary translation products of spliced mRNA. LTR, large terminal repeat. Figure credit: A. Ewing Centers for Disease Control and Prevention (CDC) B. Henderson and Arthur, National Institute of Health (NIH). C. Viral Zone, Swiss Institute of Bioinformatics. All displays from Wikipedia.

**FIGURE 2:**
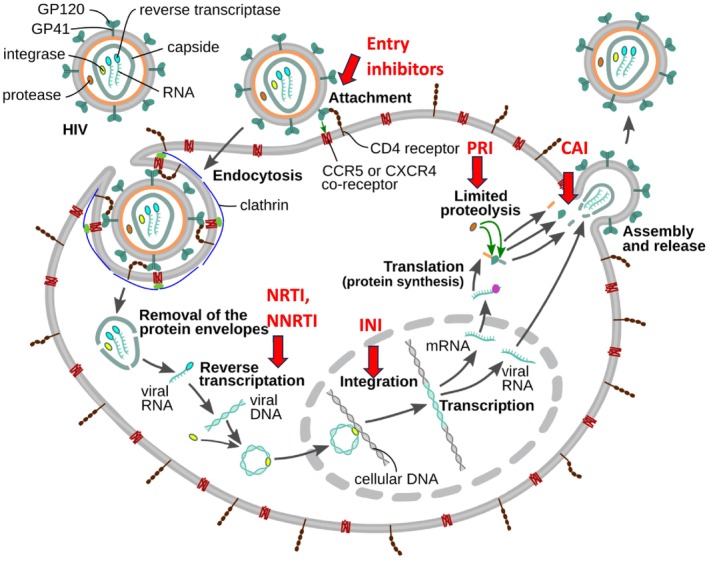
Replication cycle of HIV‐1. The scheme provides an overview from virus entry over reverse transcription of viral RNA into viral DNA, provirus DNA integration into the host genome, genome expression by transcription and translation, proteolytic processing of the viral polyproteins, viral maturation to viral release from the cell. The various targets for antivirals mentioned in the main text are indicated by red arrows and marked as entry inhibitors, nucleoside reverse transcriptase inhibitors (NRTI) or non‐nucleoside reverse transcriptase inhibitors (NNRTI), integrase inhibitors (INI), protease inhibitors (PRI) and capsid assembly inhibitors (CAI). Figure credit: scheme from T. Splettstoesser, displayed in Wikipedia.

Basic facts on the HIV‐1 virus, its morphology, protein components and genome organization, necessary for the understanding of the present article, are provided in Figure 1. Figure 2 presents an overview of the HIV‐1 replication cycle and highlights the targets of the different clinically used HIV‐1 antiviral drugs discussed below.

### 1.1 Nucleoside Reverse Transcriptase Inhibitors (NRTI)

**FIGURE 3:**
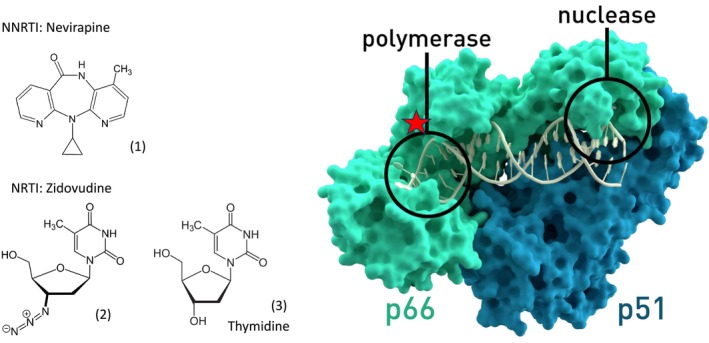
Right: Surface representation of the crystal structure of wild‐type HIV‐1 Reverse Transcriptase with its p66 and p51 subunits marked in green and blue, respectively. The active sites of polymerase and RNase are highlighted by black circles. The allosteric binding site of NNRTI is indicated by a red star. Left: At the left side are structural formula for the NNRTI nevirapine (1) and of the NRTI zidovudine (2), an analog of thymidine (3) leading to chain termination of the viral polymerase. Figure credit: RT surface T. Splettstoesser and all formulas from Wikipedia.

The first antiviral drug approved for the treatment of HIV infection was zidovudine, the prototype nucleoside analogue (Figure 3(2)). It associates with the nucleotide binding site at the active center of the RNA‐dependent DNA polymerase from HIV‐1. Since the thymidine (Figure 3(3)) analog zidovudine lacks the hydroxyl group in the 3′ position of its ribose moiety, it acts as a chain terminator during viral DNA synthesis. Zidovudine showed treatment benefits in advanced HIV disease and was approved by the Federal Drug Administration (FAD) in 1987. Oral zidovudine is well absorbed, intracellularly phosphorylated by three human kinases to the trinucleotide level and shows an in vivo half‐life, which necessitates a twice per day pill dosing. Subsequently, further NRTI were developed and approved (1991 didanosine, 1992 zalcitabine, 1994 stavudine, 1995 lamivudine, 1998 abacavir, 2001 tenofovir, 2003 emtricitabine) (Fauci and Lane 2012). These NRTI differ in efficacy, bioavailability, dosing regime, and side effects. Resistance to zidovudine developed quickly in monotherapy. Commercial preparations including zidovudine exist as 2 (combivir) or 3 (trizivir) NRTI combinations.

### 1.2 Non‐Nucleoside Reverse Transcriptase Inhibitors (NNRTI)

Nevirapine (Figure 3(1)), the first NNRTI for HIV‐1, was approved in 1996. It binds to a hydrophobic pocket of the reverse transcriptase (RT) heterodimer p66/p51 molecule. This pocket is located close to, but apart from the nucleotide binding site of RT (Figure 3 right). Nevirapine binding changes the conformation of RT, thereby inactivating the polymerase activity. It is thus an allosteric inhibitor, chemically distinct from nucleoside analogs. Nevirapine has excellent oral bioavailability and is sufficiently lipophilic to even reach the brain. A single dose is effective to prevent the transmission of HIV from the mother to the newborn when given with the onset of labor, followed by a single dose to the newborn (Fauci and Lane 2012). The NNRPI Etravirine is effective against HIV‐1 isolates that became resistant to first generation NNRTI.

### 1.3 Protease Inhibitors (PRI)

**FIGURE 4:**
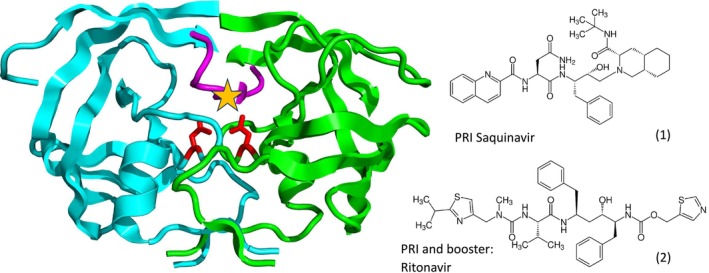
Left: The dimer structure of a HIV‐1 protease in ribbon model. The monomers are shown in green and cyan, the catalytic Asp‐25 and Asp‐25′ residues are shown in red, and Ile50 and Ile50´ residues of a hydrophobic cavity are marked in purple. The yellow star shows the binding site of ritonavir (2). The prototype protease inhibitor is saquinavir (1), a peptidomimetic drug. Saquinavir is a transition state analog of a native substrate of the HIV‐1 protease containing the dipeptides Tyr‐Pro or Phe‐Pro. Figure cedit: Boghog, from Wikipedia.

The third and largest class of approved anti‐HIV drugs are protease inhibitors. The prototype is saquinavir (Figure 4(1)), approved in 1995. The HIV protease homodimer (Figure 4 left) is encoded by the *pol* (polymerase) gene. During viral maturation, the protease liberates itself from the Gag‐Pol polyprotein, then cuts seven additional sites in this polyprotein to release nine viral proteins (Figure 1). Saquinavir is a synthetic peptide that mimics the cleavage site of the small viral protease. Saquinavir showed a poor bioavailability, but is among the best tolerated HIV‐1 protease inhibitors. Therefore, Saquinavir had to be combined with a second HIV‐1 protease inhibitor, namely ritonavir (Figure 4(2)). Ritonavir has a potent inhibitory side activity against the cellular CYP3A4 protein, a member of the cytochrome P450 protein family, involved in the biotransformation of many drugs. Ritonavir is used as a pharmacological booster to decrease the elimination of concomitantly applied drugs, thus allowing lower drug dosing, achieving less undesired side effects without compromising antiviral activity.

### 1.4 Entry Inhibitors (EI)

**FIGURE 5:**
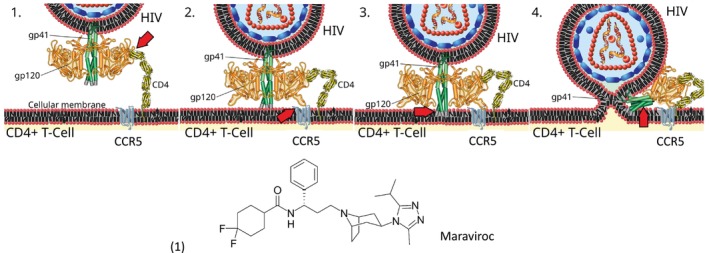
Entry of HIV‐1 into the host cell. 1. Initial interaction between gp120 SU and CD4. 2. Conformational change in gp120 allows for secondary interaction with the co‐receptor CCR5. 3. The distal tips of gp41 TM are inserted in to the cellular membrane. 4. gp41 undergoes significant conformational change; folding in half and forming coiled‐coils. This process pulls the viral and cellular membranes together, fusing them. The antiviral Maraviroc (1) can bind to the extracellular face of CCR5 (red arrow in step 2) and prevent the binding of gp120 which inhibits the subsequent entry steps. Figure credit: Mike Jones from Wikipedia.

The fourth and smallest class of anti‐HIV drugs consists of two entry inhibitors approved between 2003 and 2007. Maraviroc (Figure 5(1)) is a small chemical that binds the HIV‐1 cellular co‐receptor CCR5, a chemokine receptor. During cell attachment, HIV‐1 envelope protein gp160, consisting of gp120 SU and gp41 TM trimers, first binds via gp120 with low affinity to CD4, a glycoprotein exposed on the cell membrane mainly of T helper immune cells. The CD4 binding causes a conformational change in the gp120 which allows concomitant binding of CCR5 by gp120 leading to high affinity binding of HIV‐1 to the cell. However, CCR5 complexed with maraviroc cannot be recruited by HIV‐1 gp120, thus interrupting the cell entry process (Figure 5 top). Some HIV‐1 strains use an alternative cell co‐receptor, CXCR4, and can therefore not be inhibited with maraviroc. Enfuvirtide, a cell fusion inhibitor, is a 36‐amino acid synthetic peptide that binds to gp41 during viral attachment. Enfuvirtide binding prevents a gp41 conformational change necessary for the fusion process of the viral and cellular membranes. This fusion inhibitor must be injected subcutaneously.

### 1.5 Integrase Inhibitors (INI)

**FIGURE 6:**
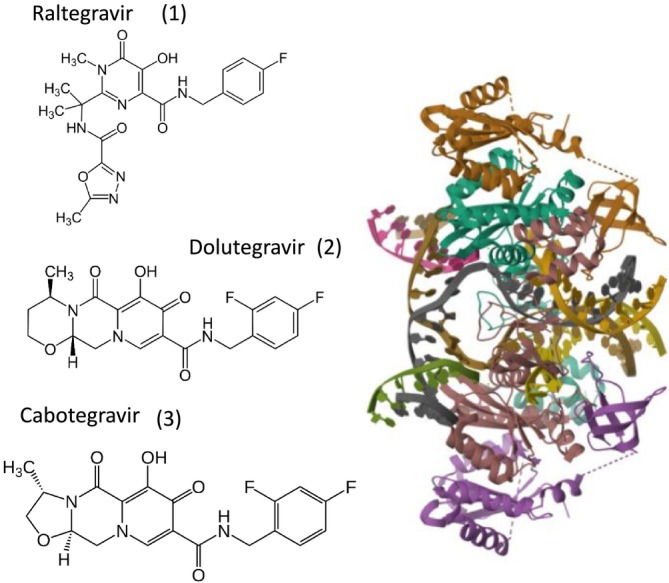
Right: Structure of the HIV‐1 Strand Transfer Complex Intasome consisting of four monomers of the viral integrase protein IN and the viral and host DNA strands. Two inner IN monomers interact at a dimer interface and are responsible for all contacts with the DNA substrates visible at the left and right center part of the intasome. The two outer monomers visible at the top and bottom of the model do not interact with each other nor with the DNA strands. At the left side are the structural formulas for the integrase inhibitors Raltegravir (1), Dolutegravir (2), and Cabotegravir (3) mentioned in the text. Figure credit: ribbon model RCSB PDB 5U1C|pdb_00005u1c, formula: Jü, Wikipedia.

The fifth class of anti‐HIV drugs is integrase inhibitors; three were approved between 2007 and 2013. The prototype is Raltegravir (Figure 6(1)). This small chemical binds to the active site of the integrase where the cut viral and cellular DNA ends are brought in close proximity for the strand transfer reaction (Figure 6 right), which mediates the integration of the viral genome into host DNA, a necessary step to viral genome expression(Figure 2). Raltegravir binding to the viral integrase causes a bending of the viral DNA end out of the active site, separating it from the host DNA end, preventing provirus integration.

Structural biology insights also led to new candidate inhibitors. In vivo, the viral integrase forms a multimer that binds the host cell transcription factor LEDGF. This complex then tethers the viral integrase to the host DNA. LEDGF introduces a loop between the two catalytic core domains of the integrase dimer. Small chemicals were designed that mimic the loop of LEDGF and hinder the viral integrase function (Jurado et al. 2013).

### 1.6 Combination Therapy Problems

**FIGURE 7:**
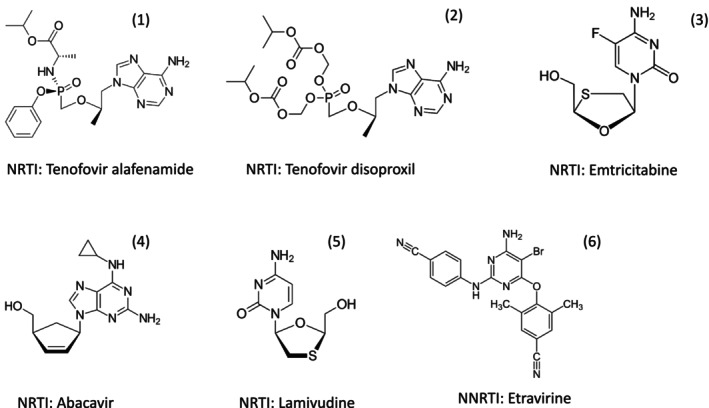
Structural formula of the HIV antivirals Tenofovir alafenamide (1), Tenofovir disoproxil (2), Emtricitabine (3), Abacavir (4), Lamivudine (5), and Etravirine (6) used in clinical trials described in the main text. Figure credit: Wikipedia.

Antiretroviral therapy (ART) is now a combination of two or more antiviral drugs (cART). Its introduction in 1995 resulted not only in a marked decline of AIDS defining conditions and a prolongation of life expectancy for HIV patients; it also resulted in suppression of viral replication thereby decreasing the rate of viral transmission (Fauci and Lane 2012). However, cART does not lead to a cure of HIV infection. A life‐long treatment with strict drug adherence is needed. This is particularly onerous for children since some cART formulations may interfere with growth and the life‐long treatment may lead to drug resistance. Clinicians searched for an optimal cART formulation. The difficulty of this task is illustrated by the complex design of the following clinical trial. Children with a treatment failure by first‐line cART are in need of a second‐line treatment formulation. Overall, 900 children from three sub‐Saharan African countries were randomised on two treatment modes: the two NRTI drugs tenofovir plus emtricitabine (FTC) (Figure 7(3)) or standard care (Musiime et al. 2025). Tenofovir exists as two prodrugs with distinct substitutions: TAF (Tenofovir alafenamide) (Figure 7(1)) or TDF (tenofovir disoproxil) (Figure 7(2)) fumarate that differ with respect to side effects. The standard care in this trial consisted of the NRTI abacavir (Figure 7(4)) plus lamivudine (Figure 7(5)) or the NRTI zidovudine plus lamivudine, depending on which first line NRTI the children had developed resistance. Ninety percent of children achieved again a reduction of viral load; the TAF‐FTC combination showed a better viral suppression and better growth than standard care. The trial design was, however, more complicated because the 900 children were also randomised on four different anchor drugs. Anchor drugs are primary, foundational medications to treat a disease. With respect to viral load reduction, growth, and cost effectiveness, the integrase inhibitor dolutegravir (Figure 6(2)) was identified as the best second‐line anchor drug.

### 1.7 Ongoing Research

Resistant development is not only a problem for clinicians but also for medical chemists. The first generation NNRTI suffers from a low genetic barrier to resistance because resistance could be achieved by a single amino acid (aa) replacement in the RT. Resistance against second generation NNRTI had also evolved under treatment but necessitated three aa mutations. These mutations defined an allosteric binding site and combined with crystallographic data of RT, medical chemists designed analogs of the pyrimidine‐based second generation NNRTI etravirine (Figure 7(6)) that filled more completely the allosteric NNRTI binding pocket mounting a higher resistance barrier while maintaining favorable pharmacological properties (Wang et al. 2025).

## 2 Antivirals for Prevention of HIV Infection in High Risk Groups

If HIV‐1 infections cannot be cured with antiviral drugs, are there possibilities of blocking HIV‐1 transmission by antiretroviral drugs used prophylactically in HIV‐negative subjects belonging to high risk groups?

Men having sex with men belong to this category. Men or transgender women from three continents, 60% reporting unprotected receptive anal intercourse and 40% practiced transactional sex were put on daily oral TDF‐FTC or placebo. After a 1‐year follow‐up of 2500 men who were HIV‐negative at baseline, 36 and 64 emerging HIV infections were observed in the TDF‐FTC and placebo arm, respectively, indicating a 44% reduction by prophylactic TDF‐FTC (Grant et al. 2010).

TAF showed improved renal and bone safety compared with TDF which induces a decline in bone density and renal impairment. In a study with 5800 mostly gay men from North America and Europe, non‐inferiority of TAF‐FTC compared to TDF‐FTC was demonstrated. TAF‐FTC was in this trial also associated with a lower rate of new HIV infection (0.16 versus 0.34 per 100 person years) and better bone mineral density and renal biomarker endpoints than TDF‐FTC, making it the preferred tenofovir prodrug (Mayer et al. 2020).

HIV‐sero‐discordant heterosexual couples are another high risk group for HIV transmission. In a trial conducted in Kenya and Uganda, 4750 such couples (in 62% of cases the male partner was seronegative) were treated with TDF, TDF‐FTC, or placebo. After follow‐up, an incidence of 0.65, 0.5, and 1.99 new HIV infections per 100 person years, respectively, were observed in the three trial arms suggesting a 67% infection reduction by HIV drugs revealing no difference between the sexes (Baeten et al. 2012).

Young heterosexual singles also belong to a high risk group when coming from a country like Botswana where up to 40% of the population in their 30s were HIV‐positive. A fifth of the 1200 HIV‐negative study participants report more than 10 lifetime sex partners. The study subjects were randomised on TDF‐FTC or placebo. Drug recipients reported more adverse events (nausea, vomiting) and a decline in bone density compared to controls but experienced less HIV infections demonstrating 1.2 versus 3.2 infections per 100 person‐years, respectively (Thigpen et al. 2012).

Based on these data, the FDA recommended TDF‐FTC for HIV prevention. However, not all trials demonstrated preventive efficiency. When 2100 HIV‐negative women from Kenya, South Africa, and Tanzania were randomised on TDF–FTC or placebo pills and followed prospectively, comparable rates of new HIV infections were observed in both trial arms, namely 4.7 and 5.0 infections per 100 person years, respectively. The women reported a median of 3 vaginal sex acts per week, a third occurred without a condom, 20% of the women were seropositive for HBV, and 40% showed bacterial vaginosis. The lack of efficiency might be due to low drug compliance: only a quarter of the participants showed pharmacological evidence for adherence to daily oral TDF‐FTC use (Van Damme et al. 2012).

Prior pharmacokinetic studies had shown lower concentrations of TDF in vaginal tissues than in rectal tissues. In fact, daily doses of TDF‐FTC were required to achieve vaginal TDF concentrations associated with HIV protection. A study addressed the question whether direct vaginal drug application conferred a better protection. About 5000 HIV‐negative women from South Africa, Uganda, and Zimbabwe were randomised into five groups, receiving orally TDF alone or TDF‐FTC or placebo, or vaginal gel with 1% TDF or placebo. About 40% of the women were human herpesvirus 2 seropositive and 40% showed bacterial vaginosis. Between 4.0 (oral placebo) and 6.8 new HIV infections per 100 person years were observed in the five trial arms with no significant difference between the groups. Again, only a quarter of the women in the oral drug group showed plasma drug levels demonstrating protocol adherence. Likewise, only half of the women from the vaginal TDF arm showed evidence for the drug in vaginal swabs, again indicating serious compliance problems (Marrazzo et al. 2015).

WHO estimated that one in 10 new HIV infections were worldwide caused by intravenous drug use. This percentage is even higher in Eastern Europe. It was thus important to assess whether prophylactic antivirals could reduce the HIV‐1 transmission rate in drug addicts. About 2400 HIV‐negative intravenous drug users from Bangkok, Thailand were randomised on oral TNF or placebo pills and followed over 4 years. The infection rate was significantly lower in the TNF than in the control arm (0.35 vs. 0.68 per 100 person years, respectively). Subgroup analysis demonstrated, however, that the protective effect was limited to women and subjects older than 40 years. Overall adherence to TNF was only 66% (Choopanya et al. 2013).

## 3 Antivirals to Reduce the Transmission of HIV to the Newborn During Pregnancy

**FIGURE 8:**
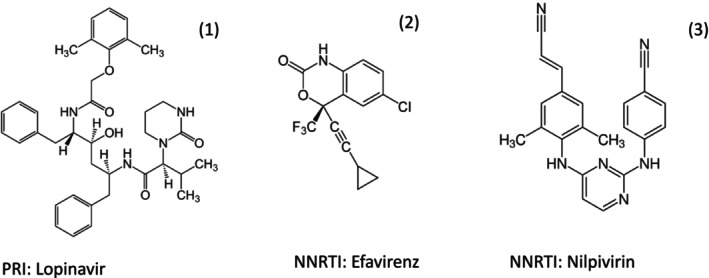
Structural formula of the HIV antivirals Lopinavir (1), Efavirenz (2), and Nilpivirin (3) used in clinical trials described in the main text. Figure credit: Wikipedia.

Epidemiological studies demonstrated that in utero HIV‐1 transmission occurs in about 25% to 30% of newborns from untreated infected pregnant women (Fauci and Lane 2012). Since each year an estimated 1.3 million HIV‐infected women become pregnant, vertical transmission of HIV‐1 is one of the drivers of the HIV‐1 pandemic. The situation is particularly dramatic in sub‐Saharan Africa as demonstrated by screening 15,000 pregnant women in Botswana where 27% of them tested in 2008 positive for HIV‐1. Substantial efforts were made to interrupt vertical HIV transmission with antivirals. Shapiro et al. (2010) compared two cART protocols in 560 HIV‐infected pregnant women from Botswana. Half of the women were treated with the NRTI abacavir, zidovudine, and lamivudine; the other half with PRI lopinavir−ritonavir (Figure 8(1)) plus NRTI zidovudine−lamivudine. An observational group of 170 pregnant women with advanced HIV disease was treated with the NNRTI nevirapine plus NRTI zidovudine and lamivudine. Suppression of the blood HIV‐1 RNA level to less than 400 copies per millilitre at delivery was achieved in more than 93% of the women and remained at 92% after 6 months of breastfeeding. Lesser viral suppression was associated with higher HIV‐1 RNA levels at baseline. Just 8 of the 709 live‐born infants were infected when tested at 6 months of age, with no significant difference in rates between the treatment groups. Six infants were infected in utero and two during breastfeeding.

Treatment recommendations are changing with incoming data provided by new clinical trials. In 2018, WHO guidelines for first‐line treatment of adults (including pregnant women) replaced the NNRTI efavirenz (Figure 8(2)) by INI dolutegravir (Figure 6(2)) in cART. The replacement was justified by new clinical observations. In 1053 mostly black adult HIV‐1 patients from Johannesburg/South Africa, dolutegravir (DTG) was compared with NNRTI efavirenz; each drug was combined with TDF‐FTC (Venter et al. 2019). The dolutegravir arm showed better suppression of blood HIV RNA levels, less discontinuation of the treatment for side effects, fewer drug interactions, and no resistance development compared to the efavirenz arm.

Also a trial in 600 HIV‐infected pregnant women mainly from Africa demonstrated a better viral suppression and less neonatal mortality in the dolutegravir than in the efavirenz arm, when each was combined with TDF‐FTC (Lockman et al. 2021).

The question arose whether these dolutegravir‐containing formulations also showed superior efficacy in an observational study with HIV‐infected pregnant women treated with many different cART formulations (Patel et al. 2022). Dolutegravir‐based ART achieved viral suppression (< 200 HIV RNA copies per ml) in 97% of patients at delivery. Among the 1724 cART treated pregnancies, only four instances of perinatal HIV transmission were observed; none occurred in the dolutegravir group.

Considering teratogenic drug effects is an important aspect when treating pregnant women. Neural tube defects were increased in newborns from pregnant mothers in Botswana when treated with dolutegravir before conception but not when treatment started during pregnancy (Zash et al. 2018).

## 4 Long Acting Injected ART for Prevention: Cabotegravir

The integrase inhibitor Cabotegravir (Figure 6(3)) is an analogue of dolutegravir. Its high inhibitory activity, low aqueous solubility, and slow metabolism allow a formulation as an injected drug. Due to its slow release from the injected nanosuspension, injected cabotegravir had in volunteers a half‐life of 20 to 50 days. In contrast, oral cabotegravir had a half‐life of 40 h. In macaques, higher injected drug concentrations correlated with higher tissue drug distribution. In a repeated low‐dose macaque infection model, untreated macaques became viremic after two viral challenge doses, while cabotegravir‐injected macaques remained uninfected. In dosing experiments, macaques became gradually infected when cabotegravir concentrations decreased. At plasma concentrations above 50 μg/mL, a 100% protection rate was observed. No proviral DNA was detected, and cabotegravir‐resistant viral mutants were not seen (Andrews et al. 2014).

In a non‐inferiority trial, 4566 participants mostly from the US and Latin America were enrolled (Landovitz et al. 2021). Participants were men having sex with men (87%) and transgender women having sex with men. The subjects were randomised on either injected cabotegravir or oral TDF–FTC. In a lead‐in phase, all participants received oral pills with the respective compounds to evaluate drug safety. After safety assessment, intramuscular injections of cabotegravir were given every 8 weeks. Oral TDF‐FTC pills were given twice daily. Placebo pills and placebo injections were given to blind the protocol. During random checks, 74% of the participants had serum tenofovir concentrations consistent with daily dosing. The participants tested HIV negative at enrolment and were followed for 153 weeks. HIV‐1 infections occurred in 52 subjects: 13 were from the cabotegravir group (incidence 0.41 per 100 person years) and 39 from the TDF‐FTC group (1.22 per 100 person‐years). The real reduction of transmission by injected cabotegravir was even greater since four infections occurred before enrolment (as indicated by using a more sensitive repeat test), five had no recent exposure to cabotegravir and three infections occurred before cabotegravir injection. At the end, only four HIV infections occurred in the fully active injected cabotegravir phase; two were with HIV strains showing a mutation to resistance in the viral integrase gene.

After the planned interim analysis, participants continued on their originally randomly assigned regimen for an unblinded additional year of follow‐up (Landovitz et al. 2023). HIV‐1 incidence in the unblinded follow‐up year was 0.82 per 100 person‐years for injected cabotegravir and 2.27 per 100 person‐years for daily oral TDF‐FTC. The risk reduction in favour of cabotegravir remained the same, but the infection risk doubled in both trial arms, which is explained by a decrease of adherence. None of the newly infected subjects in the oral group displayed a tenofovir serum concentration consistent with daily pill use. Eleven of the 17 new HIV‐1 infections in the injected group occurred more than 6 months after the last cabotegravir administration. Five showed timing irregularities for injections. Two cases displayed an integrase mutated to resistance. One case demonstrated a rapid in vivo decay of cabotegravir.

In a subsequent trial, the same protocol was applied to 3224 HIV‐1 negative women from sub‐Saharan Africa who had more than 2 sex partners, transactional sex or lived with an HIV‐infected partner (Delany‐Moretlwe et al. 2022). Adverse effects were observed with similar frequency in both groups. Weight gain of 2 kg was seen in both groups. Forty incident HIV infections were observed in a 1 year follow‐up: four in the cabotegravir group (0.2 cases per 100 person‐years) and 36 in the TDF‐FTC group (1.85 cases per 100 person‐years), indicating a nearly 10‐fold risk reduction for injected cabotegravir. Two HIV infections in the cabotegravir group occurred when the participants did not receive the injection; one was already HIV‐positive at enrolment. In contrast, all 36 infections in the TDF‐FTC group were incident infections. However, pharmacological tests revealed that only 42% of the women in the TDF‐FTC group showed tenofovir blood concentrations consistent with daily drug use.

The convenience of long acting injected ART over daily oral ART regimen motivated a clinical trial in Africa where 512 participants were randomised to injected cabotegravir plus NNRTI nilpivirin (Figure 8(3)) or continuation of the previous daily ART. The previous ART consisted of first‐line treatment with tenofovir, lamivudine, and dolutegravir which had achieved viral suppression. After 48 weeks of follow‐up, 97% of participants in both groups maintained viral suppression (Kityo et al. 2024). After 96 week follow‐up, four participants in the injected ART group, but none in the oral ART group, showed an increase in viral load. In three cases, drug resistance to both nilpivirin and cabotegravir was observed. Reinstating the previous oral ART led again to a suppression of viral replication (Kityo et al. 2025).

## 5 Long Acting Injected ART for Prevention: Lenacapavir

**FIGURE 9:**
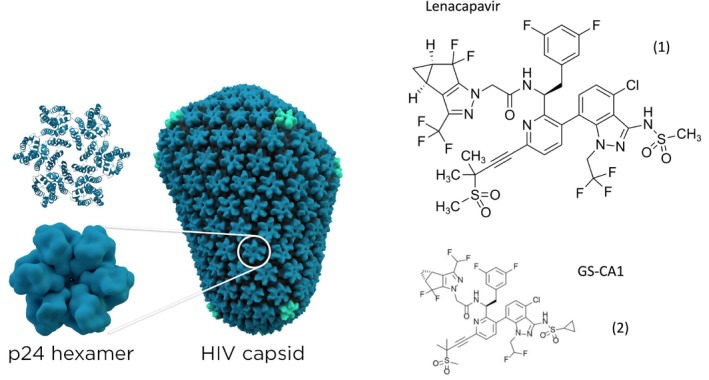
Left: The HIV capsid consists of roughly 200 copies of the p24 CA protein. The p24 structure is shown in two representations: cartoon (top) and isosurface (bottom). Right: structural formula of Lenacapavir (1) and GS‐CA1 (2). Figure credit: T. Splettstoesser from Wikipedia and Benff from Wikipedia.

The HIV‐1 capsid protein p24 is released from the Gag and Gag‐Pol polyprotein through cleavage by HIV‐1 protease (Figure 1) and capsid monomers self‐assemble into a conical capsid through protein–protein interaction. Gilead Sciences researchers screened for a small molecule that interfered with the capsid assembly process. Compound GS‐6207 (later renamed lenacapavir Figure 9(1) in clinical trials) was identified: it accelerated p24 assembly but led to malformed capsids (Link et al. 2020). This compound turned out to be a potent HIV‐1 and HIV‐2 replication inhibitor, active in picomolar concentrations while cytotoxic effects were only seen at a million‐fold higher concentration. The compound showed full activity against mutant HIV‐1 strains that had become resistant against several clinically used antiviral drug classes. GS‐6207 fits into a pocket between the N‐terminal domain of one p24 subunit and the C‐terminal domain of an adjacent p24 subunit (Bester et al. 2020) (Figure 9 left). The researchers showed that it interfered with both early and late stages of HIV‐1 replication. It suppressed provirus DNA genome integration. Upon serial HIV passage in cell culture with increasing GS‐6207 concentrations, a resistant HIV‐1 mutant was isolated that displayed an N74D substitution in p24. In healthy subjects, subcutaneously injected GS‐6207 showed a half‐life of 38 days, and reduced viral RNA loads by 4 logs in treatment‐naïve HIV‐1 patients. No resistance developed after 9 days of monotherapy. Indeed, a later small clinical trial showed that lenacapavir can reduce viral RNA blood counts by two logs in patients who developed multiple resistances against various antiviral drugs demonstrating that lenacapavir is a first in a new class of ART (Segal‐Maurer et al. 2022).

Researchers at Gilead Sciences had identified a structurally similar compound called GS‐CA1 (Figure 9(2)) with a similar binding to p24 and a comparable mechanism of inhibition (Yant et al. 2019). Several steps of viral replication were affected depending on the concentration of GS‐CA1. At 25 nM concentration, GS‐CA1 interfered with reverse transcription; at 5 nM concentration GC‐CA1 prevented nuclear import of viral cDNA and at 0.5 nM concentration, the drug reduced proviral integration without affecting the prior replication steps. In addition, HIV‐1 virions produced in the presence of GS‐CA1 contained predominantly irregularly shaped capsids. GS‐CA1 also showed antiviral efficacy in a humanised mouse model of HIV infection.

GS‐CA1 differs from GS‐6207 by a higher rate of metabolic clearance. While this is a pharmacological disadvantage, it facilitated the evaluation of time‐dependent prophylactic efficacy in a primate model. Rhesus macaques received a single injection with two different doses of GS‐CA1 or placebo, followed by weekly repeated challenges with simian–human immunodeficiency virus (SHIV) of increasing doses (Vidal et al. 2022). The researchers determined blood levels of GS‐CA1 and the time to viremia. The lower 150 mg/kg dose significantly delayed viremia compared to controls (100% infected) for 8 weeks (20% remained uninfected), while the higher 300 mg/kg dose delayed infection even further with 60% of the animals showing no sign of infection. Infections occurred when the plasma GS‐CA1 level fell below 100 nM. The protocol allowed to assess the level of GS‐CA1 needed for full protection and to look for resistant viral mutants during the wash‐out phase of this monotherapy. No resistance developed during the 24‐week observation period.

Lenacapavir showed striking clinical efficacy. About 5300 adolescent girls and young women from South Africa and Uganda were enrolled into a phase 3 randomised, double‐blinded, placebo‐controlled trial (RCT) of HIV‐1 prevention (Bekker et al. 2024). The participants were HIV‐1 negative at enrolment and received either subcutaneously injected lenacapavir every half year or daily oral TAF‐FTC or TDF‐FTC pills. The subjects were followed over a year for incident HIV infections. No infections were observed in the lenacapavir arm compared with 39 infections (corresponding to 1.69 infections per 100 person years) in the TDF‐FTC and 2.02 infections per 100 person years in the TAF‐FTC arm. Most participants in the two oral drug groups had low drug adherence: more than four doses of tenofovir per week were detected by blood tests in only half of the participants shortly after enrolment. Adherence eroded over the test and decreased to about 10% after a year. The majority of subjects who experienced an HIV‐1 infection had low or no detectable tenofovir concentrations in the blood at the moment of infection. In parallel, the researchers conducted an observational survey in 8400 adolescent or young women. They observed an HIV‐1 infection prevalence of 6.2%; a fifth of the infections were diagnosed as recent infections, suggesting an infection rate of 2.41 per 100 person years in this reference population group. The oral TAF‐FTC arm did thus not differ significantly from the background HIV‐1 incidence rate. There was no meaningful difference in HIV‐1 incidence between the oral TDF‐FTC and TAF‐FTC arms. Only the lenacapavir arm showed a significant and indeed dramatic reduction in incident HIV‐1 infections. Adverse events were low grade injection site pain. Headache, urinary, and urogenital infections were observed with similar frequency across the three trial arms.

Next, 3200 male participants (75% gay, 15% bisexual) mostly from America (Brazil, US, Peru) were enrolled into a phase 3 RCT (Kelley et al. 2025). The participants received a twice yearly subcutaneous injection of lenacapavir or daily oral TDF‐FTC pills. Two incident HIV‐1 infections were observed in 2183 lenacapavir‐injected subjects (corresponding to 0.1 infections per 100 person years). Both subjects showed the expected lenacapavir plasma concentrations, but the infecting HIV‐1 strain showed in both cases the N74D capsid resistance mutation. In the TDF‐FTC arm, the infection rate was 0.93 per 100 person years; all infected subjects showed low or no oral drug adherence. Overall, the oral drug adherence was relatively high: it was 82% after 2 months, and 62% after a year, which might explain the lower HIV infection rate in TDF‐FTC groups in men from America than in women from the African trial. The background HIV‐1 infection rate in this population was determined by HIV‐1 screening in 4800 subjects. The diagnosis of recently acquired HIV infection yielded a spontaneous infection rate of 2.37 per 100 person years. This parallel screening avoided the ethical dilemma of running an untreated placebo group. The efficacy of injected lenacapavir in comparison with the background was 96% and 89% when compared with oral TDF‐FTC. Adverse events during the trial were rectal chlamydia and oropharyngeal and rectal gonococcal infections, which occurred with similar frequency in both groups. The subcutaneous injections resulted in palpable but not visible nodules at the injection site (greater in the lenacapavir than in placebo injections) that resolved over time. The authors noted that more than 99% of the participants in the lenacapavir group did not acquire HIV‐1 infection, despite the high levels of sexual exposure, use of drugs in conjunction with sex (‘chemsex’), and sexually transmitted infections.

## 6 Chances to Stop the HIV Pandemic: Cost and Political Considerations

The global rollout of PreExposure Prophylaxis (PrEP) with oral cART began in 2015 but by 2020 fewer than 1 million people in developing countries were on oral PrEP and drug adherence was low when tested in clinical trials. The long‐acting injectable drugs are a potential game changer against the epidemic spread of HIV‐1. African women prefer injected over oral drugs because it is a more discrete application and resembles injected contraception methods. However, the cost of the interventions is a major hurdle to widespread use. ViiV Healthcare has priced injectable PrEP cabotegravir at USD 22,000 for a year's supply in the United States. Approximately 15,000 people have started cabotegravir for PrEP worldwide; for cost but also drug approval most users are from the US (Cantos et al. 2025). ViiV Healthcare has pledged to provide cabotegravir at nonprofit prices to the poorest countries, but has not yet specified the price tag (Green 2022).

In a recent cost‐effectiveness analysis from South Africa, it was assumed that injectable cabotegravir averted 15 to 28% more new HIV‐1 infections compared with an oral cART scenario. The cost per one HIV‐1 infection averted with the best oral cART was USD 6000 (the exact value depends on adherence to the oral drug use). The cost per cabotegravir injection needed to be less than twice that of a 2‐month supply of TDF‐FTC to be cost‐effective. This would mean about USD 100 for an annual treatment with cabotegravir in low and middle income countries (LMIC) (Jamieson et al. 2022), 200‐fold lower than the current list price for cabotegravir.

The current lenacapavir list price for HIV treatment is between USD 25,000 and 40,000 for one person year in different high‐income countries. FDA approved its use for treatment of patients with multi‐resistant HIV‐1 in 2022 and for PrEP in June 2025. The Senior Vice President of Gilead Sciences, a prior prominent HIV researcher, justified this price by the two decades of HIV‐1 research investments by his company. He admitted that scientific data on their own will not end the epidemic; access and uptake are essential for a public health impact (Baeten 2025).

Pharma companies need a decent return on investment (ROI) as capitalistic companies. ROI and affordability of efficient drugs for a population that needs it most—and for infectious diseases, this is frequently the population from LMIC—presents thus an economic and public health dilemma. Gilead's approach is to enable generic manufacturers to produce its medicines at large scale for low‐ and lower‐middle‐income countries ahead of patent expirations through direct voluntary licensing. Gilead started this process within weeks of obtaining the lenacapavir clinical results. Volume is also a problem: ramping up the manufacturing capacity to produce the drug for 2 million people would need 2 years. Gilead assured that the current US list price for lenacapavir will not be the reference point for PrEP pricing (Baeten 2025).

Injected drugs such as lenacapavir have the potential to curtail the spread of the HIV‐1 pandemic. Whether such drugs will live up to their potential will depend on economic and political decisions. Academics have calculated the cost of production of lenacapavir (Hill et al. 2024). The lenacapavir active pharmaceutical ingredient (API) is currently exported from India for USD 60,000/kg on a 1 kg scale. This price could be halved when committing to a command for 1 million treatment years. Including formulation steps, a 30% profit margin and 27% tax on profit (but not accounting for ROI for the parent company), a generic injected lenacapavir could be produced for about USD 100 per person year. Voluntary licensing and multiple suppliers are, according to these calculations by academics, required to achieve these low prices.

However, there are also political hurdles for a worldwide effort to limit the spread of HIV‐1, not to speak of ending the HIV‐1 pandemic. To illustrate this point: The US Agency for International Development (USAID) is the largest funding agency for humanitarian and development aid worldwide. Epidemiologists calculated that USAID funding, which targeted mostly Africa, was associated with 15% reduction in age‐standardised all‐cause mortality or even a more impressive 32% mortality reduction in under‐five year‐old children. US funding between 2001 and 2021 prevented 91 million deaths (including 30 million deaths in children). USAID financial support had the highest impact on the reduction of mortality from HIV‐1 infections over other infectious diseases (e.g., tuberculosis or malaria) (Cavalcanti et al. 2025). In 2023, the USA accounted for 43% of all government funding donated by countries to the humanitarian system, up from about 39% a decade earlier. The US President's Emergency Plan for AIDS Relief (PEPFAR), launched in 2003 had invested an accumulated amount of over USD100 billion in the global HIV/AIDS response. In 2023, 60% of PEPFAR's bilateral HIV‐1 assistance was implemented by USAID. This beneficial effect of US support for public health is not likely to perdure. The US administration has ordered the withdrawal from WHO, taking effect in January 2026, and ordered the US CDC to stop its collaboration with WHO. HIV clinics in Africa that were supported by PEPFAR started turning away clients. On March 10, 2025, it was announced that 83% of the programs run by USAID would be cancelled (Cavalcanti et al. 2025) and there were reports that USAID would see its staff cut from 10,000 to less than 300 (Burki 2025). Forecasting models predicted that the current steep US funding cuts in USAID alone could result by 2030 in more than 14 million additional all‐age deaths, including 4.5 million deaths in children younger than 5 years, with a major share caused by AIDS casualties in Africa (Cavalcanti et al. 2025). This projected boost of the AIDS pandemic—should the cuts become reality‐ could also have spillover effects for high income countries. Less HIV drugs will translate into more circulating HIV‐1 virus which will also create more viral mutants and this might compromise the efficacy of currently available HIV‐1 antivirals. Sadly, pandemic viruses know no frontiers in a connected world. Loosing control of HIV‐1 in Africa might have worldwide public health impacts.

The scientific success of injected antiviral drugs raised a final question. Do we still need an HIV vaccine which has so far eluded the research community when we have a highly efficient injected prophylactic drug? One could argue that lenacapavir can be compared to a vaccine that needs a boost every 6 months, which is not very different from the timing of the yearly new influenza virus or coronavirus vaccines. Technically, economically, and ethically, the availability of an effective prophylactic drug will complicate conducting vaccine trials. However, it might be safe to not put all eggs in a single basket and pursue HIV‐1 vaccine research in the future despite the promises of injected antivirals.

## Author Contributions

**Harald Brüssow:** conceptualization, writing – original draft, investigation.

## Funding

The author has nothing to report.

## Conflicts of Interest

The author declares no conflicts of interest.

## Data Availability

All reported data in this Opinion article were extracted from the publications quoted in the Reference list.
